# The Advantages of Nitrous Oxid-Oxygen Anesthesia, Especially in Connection with Ultimate Recovery*Read before the New York Academy of Medicine, Section on Surgery, Nov. 18, 1910.

**Published:** 1911-04-15

**Authors:** Raymond C. Coburn

**Affiliations:** New York


### THE ADVANTAGES OF NITROUS OXID-OXYGEN ANESTHESIA ESPECIALLY IN CONNECTION WITH ULTIMATE RECOVERY*

RAYMOND C. COBURN, M.A., M.D.
NEW YORK.

As the aim of the surgeon is to record a "successful operation," not only from a scientific point of view, but from the patient's standpoint as well, the selection and administration of the anesthetic becomes more and more a matter for consideration as this branch of medical science is developed.

Until recent years the dangers of the anesthetic were considered solely those occuring on the operating-table, but laboratory investigation has established, beyond the possibility of a doubt, that a percentage of surgical mortality, heretofore considered to be due to the patient's condition, effects of the operation, etc,. is sometimes due directly to the effects of the anesthetic, even though death occurs remotely from the time of the operation. Careful and extended clinical observations on nitrous oxid-oxygen anesthesia in several thousand cases, by some of the most talented surgeons of this country, completely verifies this conclusion.

*Read before the New York Academy of Medicine, Section on Orthopedic Surgery, Nov. 18, 1910.

The use of nitrous oxid as an anesthetic agent presents an interesting and a very peculiar history, which I shall not attempt to detail here. To Andrews, of Chicago, belongs the credit of discovering that by the addition of the proper amount of oxygen, the asphyxial symptoms of nitrous oxid are prevented without destroying its anesthetic properties.

Since this discovery was made, over forty years ago, nitrous oxid-oxygen has slowly, intermittently, but very persistently gained recognition until to-day it occupies the meritorious position of being endorsed by one of the largest and greatest scientific socities of the world, as being all effects considered, the best means for producing anesthesia that is known.

There is a prevalent idea that nitrous oxidoxygen is better adapted for minor surgery and short operations than for major work. This is an erroneous impression, however, for the longer this anesthesia is maintained, the better it becomes in every way. I myself have administered it throughout practically the whole range of operative procedure from a simple incision to difficult and complicated operations lasting over four hours, which is the longest period, as far as I know, where this anesthetic ha ever been continuously administered. Dr. Frederick Hewitt, of London, a leading authority on anesthetics, say :

There is no form of anesthesia at present known which is so devoid of danger as that which results from nitrous oxid when administered with a sufficient percentage of oxygen to prevent all asphyxial complications.

The Anesthesia Commission of the American Medical Association reported at its last session in St. Louis as follows:

As a routine anesthetic nitrous oxid has a peculiar value, . . . (and) in the hands of highly skilled anesthetists the method is the be t yet devised.

These statements are corroborated by such practical surgeons as Crile, Meyer, Kelley, Halsted and Bevan, who collectively have certainly used it in thousands of cases, and are therefore in a position to express an opinion of value as to its superior influence toward ultimate recovery.

There is much less surgical shock and postanesthetic nausea and depression with nitrous oxid than with ether or chloroform; the patient regains consciousness very quickly and, being less depressed, recovers from the effects of the operation sooner and better.

Hamburger and Ewing, who have made an extended laboratory investigation of the effects of the narcosis produced by nitrous oxid, chloroform and ether on the blood, and whose findings are published as a part of the preliminary report of the Anesthesia Commission of the American Medical Association, say, as regards ether:

The color index shows a rather constant drop, starting immediately after anesthesia and reaching its lowest point on the fifth and sixth days. This would indicate a relative loss of hemoglobin per cell, and again is unlike nitrous oxid results, in which the only sign of a low color index is found immediately after the anesthetic mask is removed, and which is completely gone in two hours.

The volume index likewise shows an immediate loss, which is most marked in twenty-four hours, and again on the fifth to seventh days. In nitrous oxid readings the percentage volume remained unchanged throughout.

According to these investigators so short an anesthesia as fifteen minutes with ether causes a definite and demonstrable anemia, and such an effect as this, which does not begin to improve till after the fifth day, must certainly retard convalescence even in favorable cases, and in critical cases may be the determining factor in producing a fatal termination.

Chloroform produces even more destructive changes on the blood than does ether, while its degenerative action on the liver has been mentioned by so many writers that this serious and remote effect needs merely to be mentioned here.

For some time it has been recognized that ether increases the toxemia arising from infection. Recent investigation shows that this is due to the fact that ether impedes the functional activity of the leukocytes: that is, it lessens the patient's resisting power against the infection, whereas nitrous oxid exerts no such injurious effect on the blood-cells, and consequently does not lower the patient's immunity which naturally exists. In serious cases of infection, as well as in the "borderland" cases in general, the difference in ultimate recovery under the two anesthetics is strikingly shown.

Numerous surgeons have advised against the use of ether in tuberculous conditions, as, in their experience, it causes new foci of infection to develop. This, however, is only a particular instance of the general effect produced, for, no matter what the infection may be, the toxic effect of ether on the leukocytes lessens the efficiency of Nature's effort to combat the invading host.

As is well known, ether is an irritant to the respiratory mucous membrane and to the kidneys, whereas nitrous oxid is without deleterious effect on these tissues, and is therefore strongly indicated as the anesthetic agent whenever these structures are involved, no matter what the operation may be, as in tuberculosis, pneumonia, bronchitis, laryngitis, nephritis, diabetes, pyelitis, and a host of other inflammations and infections of the respiratory and genito-urinary tracts. In fact, when you take into consideration the lessened amount of surgical shock and of postoperative discomfort and depression, the absolutely non-irritant action on the mucous membranes of the tracts involved in its administration and elimination, and the blood elements left unimpared, its range of usefulness is so broad that it should be the anesthetic of choice, except in instances in which it is specially contra-indicated. Besides, when this anesthetic is used, there is less liability of infection occuring during, or subsequent to the operation, inasmuch as the patient is less depressed, and natural immunity undisturbed. These are practical points which every operator should carefully consider, as they are important elements, no matter how trivial each may at first appear, when considered separately, for, taken collectively, they most certainly affect the postoperative condition in every case.

It is readily granted that in the hands of the skilled anesthetist there is very little immediate danger in the use of the other anesthetics, and, that, in such hands, the immediate mortality is very low indeed; but is it fair to conclude that the remote effects, much more subtle in their influence, and therefore much more difficult of detection, are consequently *nil*? Take, for instance, a case of grave infection; one of the other general anesthetics is faultlessly administered, but the surgeon's mortality is increased, and every surgeon of wide experience encounters some such cases. In the light of this research can it be said that the anesthetic did not contribute to the fatal result, or that the patient might not have been saved had a less toxic and less depressing agent, and one which did not lessen the patient's natural resisting power, and decrease the life-sustaining function of the blood-cells, been used? It is not contended that this form of anesthesia saves all patients of this class but, in the language of Crile, "Not a patient showed the rapid march to fatality immediately following the operation which occasionally follows ether." Why the conspicuous absence of that "rapid march to fatality"? Simply because the patient is given a new and firmer hold on life, enabling some to recover that otherwise would simply increase the mortality. I very readily assent to the observation of Gatch that "very sick patients, with a rapid pulse and quick, shallow respiration, actually seem benefited by this form of anesthesia." That is, such patients will, during an operation under this anesthesia lasting for forty-five minutes to an hour, manifest a distinct improvement in the rate and quality of the circulation and respiration, both during and subsequent to the operation, the effect being quite permanent, as it is not a mere stimulant.

From the patient's point of view the lessened amount of postoperative discomfort alone is sufficient to call for this anesthetic, as he usually dreads the subsequent nausea, vomiting and depression more than the effects of the operation itself. Besides, when a patient can be told that he is simply to be given "laughing-gas" it allays to a very great extent the fear and dread of the anesthetic, and many patients will submit to operations under this agent who would otherwise refuse all operative intervention.

It is a matter of no little comfort to the patient and to his family to know that complete consciousness returns so quickly after the operation is completed, and especially is this so, when, as in the majority of cases, there is freedom from postnarcotic discomfort, and a pleasant impression is left on the mind by the "laughing-gas." For a patient to be returned from the amphitheater to his own room completely conscious, smiling and talking to his nurse and friends, even after a prolonged operation, presents a different picture from that usually seen with other anesthetics, and gives a distinct advantage in convalescene. I cite just one case to illustrate: Dr. X. of a neighboring state was anesthetized for a rectal operation, necessarily requiring deep anesthesia. Within a very short time after the administration of the anesthetic was stopped he opened his eyes and said with a smile to those in attendance: "Well" if you folks had as good a time as I did, you certainly enjoyed this operation." He had had a dream about being at a big fire. Some time afterwards he said in a private letter to me: "You simply make anesthesia a pleasure." To have an intelligent patient, and especially a practitioner of wide experience, consider the taking of the anesthetic as a genuine pleasure is certainly valuable testimony to the great advantage offered by this anesthetic.

It is almost imperative to use a preliminary hypodermic injection of morphine and atropin about one-half hour before the time of the operation, as this medication aids very materially in securing muscular relaxation. Besides, the patient recovers from this anesthesia so quickly that it is usually indicated for the postoperative pain alone. When it is used the patient's nerves are quieter and he approaches the operation with more confidence and less fear—an important element in every case. In that class of patients or operations in which it is difficult to overcome muscular rigidity the addition of a very small amount of ether—not sufficient to give the ether after-effects—in conjunction with the nitrous oxid and oxygen will secure the necessary relaxation. Used in this way, nitrous oxid-oxygen is a pleasant anesthetic for even the most fastidious patient, fulfils the requirements of both minor and major surgery, and produces the lightest and least toxic general anesthesia yet discovered.—*Journal A. M. A.*

